# Prosthetic Emergence Angles and Implant Outcomes: A Retrospective Study With at Least 5 Years of Follow‐Up

**DOI:** 10.1111/cid.70127

**Published:** 2026-02-25

**Authors:** Paolo Dellacasa, Lucia Schiavon, Maria Menini, Paolo Pesce, Cristiano Tomasi, Eriberto Bressan

**Affiliations:** ^1^ Private Practice in Genova Genova Italy; ^2^ Clinic of Reconstructive Dentistry, Center of Dental Medicine University of Zurich Zürich Switzerland; ^3^ Department of Neurosciences, Dentistry Section University of Padova Padua Italy; ^4^ Division of Prosthodontics and Implant Prosthodontics, Department of Surgical Sciences (DISC) University of Genova Genova Italy; ^5^ Department of Surgical Sciences (DISC) University of Genova Genova Italy; ^6^ Department of Periodontology, Institute of Odontology, Sahlgrenska Academy University of Gothenburg Gothenburg Sweden

**Keywords:** dental implant, dental implant abutment design, emergence angle, emergence profile, titanium abutment

## Abstract

**Objectives:**

The influence of prosthetic emergence angles on peri‐implant tissue stability has become a topic of growing interest. While wide emergence angles have been associated with increased marginal bone loss (MBL) and peri‐implantitis, available evidence is limited and largely based on studies using prefabricated abutments. This retrospective study aimed to evaluate the impact of customized implant‐supported fixed dental prostheses (iFDPs) emergence angles on peri‐implant MBL and peri‐implant health after a minimum of 5 years of follow‐up.

**Materials and Methods:**

Patients rehabilitated with single or multiple iFDPs between 2010 and 2019 were retrospectively screened. Inclusion required a minimum follow‐up of 5 years. Clinical parameters and radiographic marginal bone loss were assessed. Emergence angles (mesial, distal, buccal, palatal/lingual) were measured and categorized as < 40°, 40°–59°, or ≥ 60°. Multivariable regression models adjusted for smoking and follow‐up duration analyzed associations between emergence angles, MBL, and peri‐implantitis.

**Results:**

Fifty‐two patients with 112 implants met the inclusion criteria. Mean emergence angles were: mesial 43.9°, distal 40.6°, buccal 49.5°, and palatal 45.8°. Overall, peri‐implant tissues remained stable, with mean bone loss values ≤ 0.8 mm and mean PPD 1.8–3.9 mm over time. No significant correlation was found between emergence angle categories and MBL.

**Conclusion:**

Prosthetic emergence angle did not significantly influence peri‐implant bone loss or peri‐implantitis when customized abutments were used. Individualized abutments might mitigate risks associated with wide emergence angles.

## 1 Introduction

Implant‐supported fixed dental prostheses are a reliable and safe treatment option for the rehabilitation of total and partial edentulism, with a 97.1% success rate [1, 2, 3, 4, 5]. A key factor in ensuring the long‐term success of dental implants is the stability of peri‐implant marginal bone [6]. While some bone resorption is considered physiological during the first year after implant loading [7], many factors can lead to pathological peri‐implant marginal bone loss (MBL) over time. The most common cause of MBL is peri‐implantitis, defined as inflammation of the peri‐implant tissues triggered by plaque accumulation [7]. Other contributing factors include implant misplacement, systemic health conditions, history of periodontitis, and inadequate soft tissue thickness [8]. Recent investigations have also highlighted the potential influence of prosthetic factors, such as abutment height, implant‐abutment connection, and prosthetic emergence angle, on MBL [9, 10, 11].

Among these, the impact of implant‐supported fixed dental prostheses' (iFDPs) emergence angle on peri‐implant health has become a trending topic in the scientific literature. The prosthetic emergence angle is defined as the angle between the average tangent of the transitional contour relative to the long axis of the dental implant [6, 12]. From a clinical standpoint, it is well understood that suboptimal prosthetic designs—especially those that hinder effective cleaning—can contribute to peri‐implant disease [13]. Despite this knowledge, there are currently no evidence‐based guidelines for the design of prosthetic frameworks of iFDPs [14]. Additionally, the customization of the transmucosal segment and the resulting emergence angle varies greatly depending on the clinical situation, making it challenging for clinicians to follow a standardized approach.

Previous research has suggested that emergence angles greater than 30° or 40° could contribute to marginal bone loss (MBL) and increase the risk of peri‐implantitis [15, 16, 17]. A recent preclinical study has supported this theory, showing a trend of increased MBL over time with restorative angles exceeding 40° [11]. This is crucial, since MBL is considered a critical factor in implant success [18]. Along with emergence angle, the emergence profile also plays a crucial role in maintaining peri‐implant tissue health and stability. A convex emergence profile has been associated with a significantly higher risk of buccal bone recession [14]. Furthermore, implant‐supported crowns with > 30° emergence angles and convex profiles have been associated with increased MBL [16].

However, it is interesting to note that there is no consensus on how prosthetic contour influences MBL. A recent systematic review [19] indicated that angles higher than 30° might be linked to a higher prevalence of peri‐implantitis or MBL. In contrast, another systematic review [20] found no significant differences in clinical outcomes between prosthetic emergence angles above or below 30°. It must be noted that both these systematic reviews were able to include only cross‐sectional or retrospective studies and included a limited number of clinical studies, highlighting a lack of clinical evidence on the topic. Furthermore, the great majority of the available studies focus on Titanium bases standard abutments [11, 15, 16, 21]. Despite their widespread use, Ti‐base abutments cannot accommodate site‐specific anatomical variations [15, 22, 23]. While customized abutments improve aesthetic integration and customization of the transmucosal path [24], they imply increased costs and more complex implementation, and their long‐term impact on peri‐implant bone stability remains unclear. This knowledge gap necessitates investigation into the relationship between customized emergence angles and profiles and long‐term peri‐implant tissue preservation.

Therefore, the aim of the present observational retrospective study was to evaluate the effect of different customized iFDPs emergence angle on clinical parameters and radiographic marginal bone levels at implants.

## 2 Materials and Methods

The present retrospective observational study was conducted in accordance with the Helsinki Declaration and was approved by the local Ethical Committee of the University of Genova (CERA n. 2024/35).

### 2.1 Patient Selection Process

All the patients restored with single or multiple partial implant‐supported fixed dental prostheses (iFDPs) between July 2010 and December 2019 have been retrospectively screened. Only patients with a minimum follow‐up of 5 years have been included. All the included patients were informed about the objectives of the study and gave their written consent.

### 2.2 Implant Placement

All the clinical procedures and follow‐up examinations have been performed in a private practice setting in Genoa, Italy, by one experienced periodontist (PD). Before surgery, patients received a clinical and radiographic evaluation, and only patients who had good oral hygiene (full mouth plaque index < 25%) [25] and adequate control of inflammation (BOP < 25%) [26] were considered eligible for dental implants. Panoramic and periapical radiographs were used as a first‐level x‐ray examination. If a more detailed assessment of bone width and height was needed, cone beam computed tomography (CBCT) was performed as a second‐level x‐ray examination. After a full‐thickness flap elevation, freehand implant placement was performed, following the manufacturer's recommendation. If needed, simultaneous hard tissue grafting was performed with autologous bone and/or deproteinized bovine bone mineral (Bio‐Oss, Geistlich Wolhusen, Switzerland) covered either with platelet rich fibrin (PRF) or a resorbable collagen membrane (Bio‐Gide, Geistlich Wolhusen, Switzerland). Primary intention healing was obtained through single interrupted 4.0 nonresorbable sutures (Glycolon, RESORBA Medical GmbH, Nürnberg, Germany). Transmucosal healing was chosen when primary stability was obtained. When simultaneous bone regeneration was performed or primary stability was not obtained, submucosal healing was chosen.

### 2.3 Prosthetic Procedure

**FIGURE 1:**
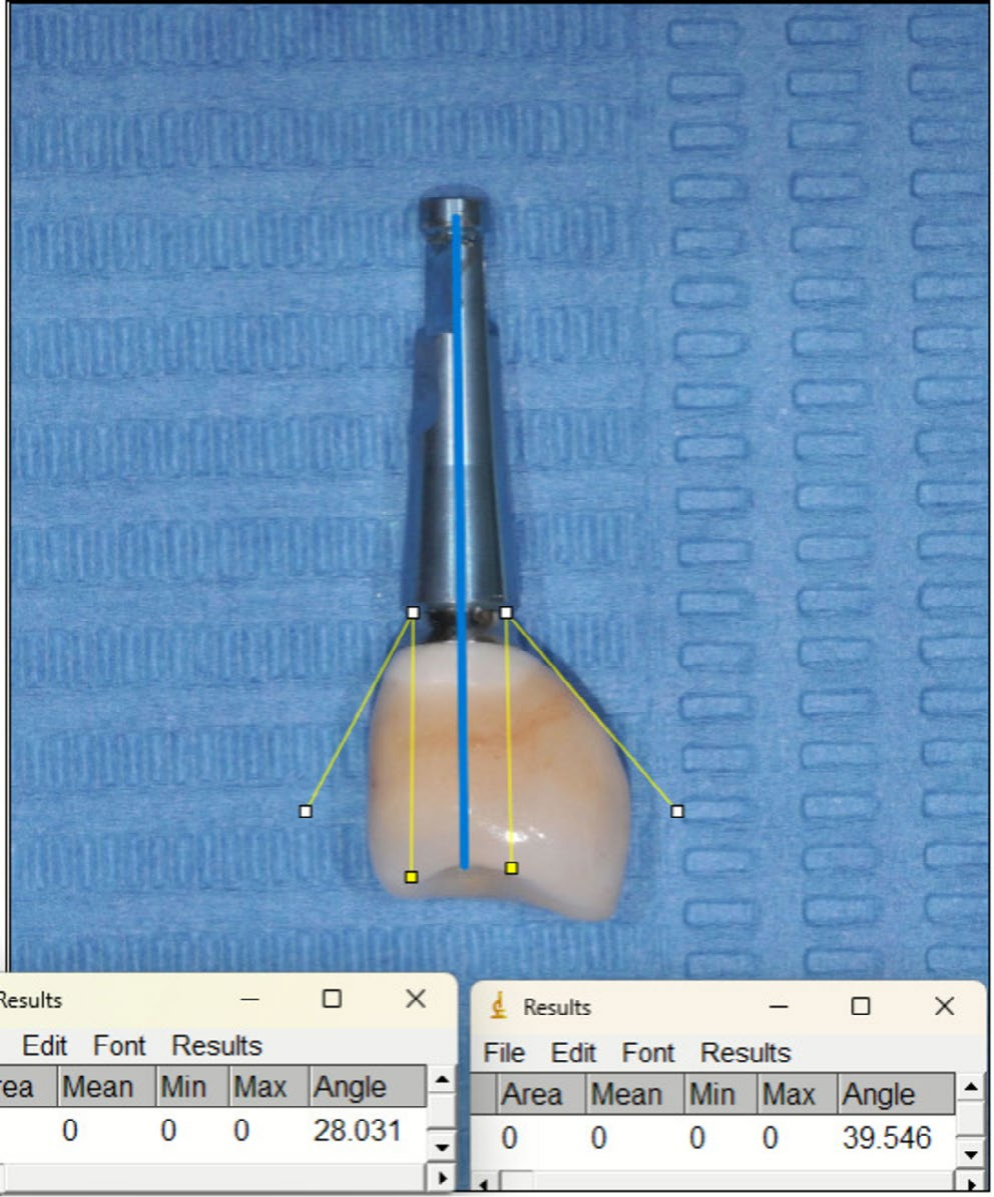
Example of prosthetic emergence angle assessed extra orally, before crown delivery (buccal and lingual/palatal).

Three months after implant placement, an implant level analogical open‐tray impression was taken (Impregum Penta Soft, 3 M). Two to four weeks after implant impression, final screw‐retained metal‐ceramic implant‐supported FDP were delivered, following the manufacturers' instructions. All implants included in this study were restored following the platform‐switch concept. Occlusal contacts were adjusted using an 8‐μm shim‐stock foil (Arti‐Fol; Dr. Jean Bausch GmbH & Co. KG) for pull‐through and 20‐μm articulating paper for determining contact points [27]. Buccal and palatal emergence angles were evaluated by analyzing standardized extraoral photographs of the prosthetic restorations positioned on an implant analog before crown insertion (Figure 1). All prosthetic photographs were acquired under standardized conditions using a Nikon D300 digital single‐lens reflex (DSLR) camera (Nikon Corporation, Tokyo, Japan) equipped with a Nikon AF‐S VR Micro‐Nikkor 105 mm f/2.8G macro lens. Images were captured at 2.5× magnification, with an aperture of F32, from a fixed distance of 0.8 m to ensure consistency across all photographs. Baseline for clinical and radiographical parameters was at crown delivery. Each patient received an individualized maintenance program, consisting of regular dental hygiene sessions (ranging from 3 to 12 months) and clinical and radiographical follow‐up during the entire study period.

### 2.4 Radiographic Evaluations

**FIGURE 2:**
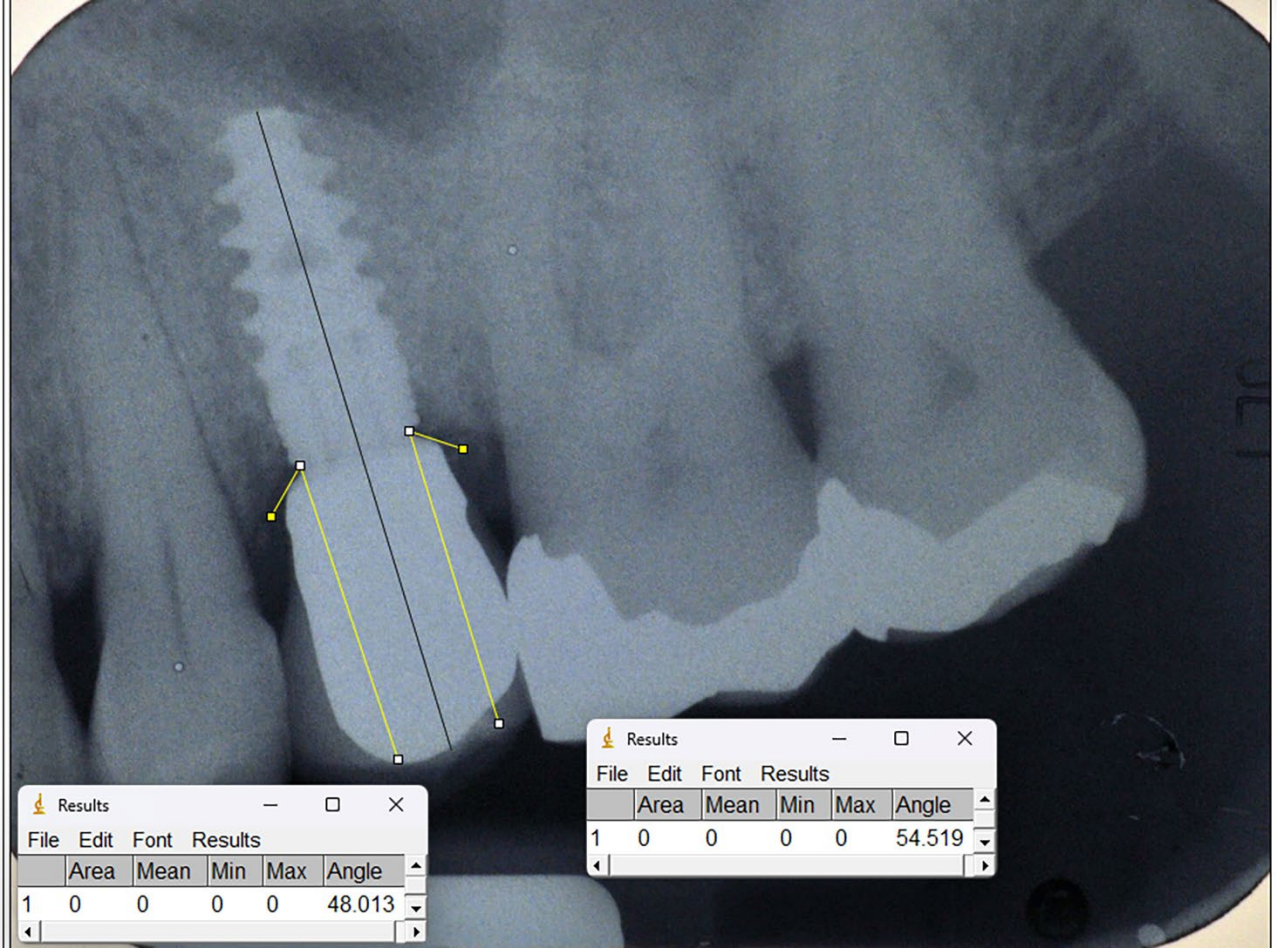
Example of prosthetic emergence angle assessed on the radiograph (mesial and distal).

Follow‐up periapical radiographs were obtained using a parallel technique with Rinn holders and retrospectively collected. X‐rays were then imported to an open‐source software (ImageJ 1.53; National Institute of Health, Bethesda, USA). For each implant‐supported FDP, mesial and distal emergence angles [12] were measured as described in Figure 2. The same software was used to measure marginal bone level at the mesial and distal aspects of each implant, both at baseline and at the most recent follow‐up period. All the radiographic measurements have been performed by a single experienced clinician (PD).

### 2.5 Clinical Evaluation

For each implant, clinical parameters were measured at four sites by a calibrated examiner, using a North Carolina Probe (UNC 15, Hu‐Friedy, Chicago, USA):

– Probing pocket depth (PPD) [7]
– Bleeding on probing (BOP) (%)
– Plaque index [13] (%)

Peri‐implant mucositis and peri‐implantitis were assessed as described in the consensus report of the 2017 World Workshop on the Classification of Periodontal and Peri‐Implant Diseases and Conditions [7, 28].

### 2.6 Data Analysis

Data analysis was performed using Stata version 17.0 (StataCorp LLC, College Station, TX, USA). Emergence angles were categorized into three groups (< 40°, 40°–59°, ≥ 60°) for analysis. These thresholds have been selected based on previous preclinical [11] and clinical [29] studies, which reported a positive correlation between prosthetic angles wider than 40° and 60° and increased MBL. Marginal bone loss (MBL) was calculated as the difference between radiographic measurements at loading‐to‐1‐year (MBL_1y), loading‐to‐final visit (MBL_final), and 1‐year‐to‐final visit (MBL_1yfinal).

Multivariable linear regression models evaluated associations between emergence angles and MBL, adjusting for smoking status and follow‐up duration. Cluster‐robust standard errors were used to account for multiple implants per patient. Probing pocket depth (PPD) measurements were analyzed at all timepoints, and correlation with emergence angle was tested.

Multivariable linear regression models examined the relationship between final bone loss and pocket depth, emergence angle categories, and their interaction, adjusting for age, sex, and smoking status, with standard errors clustered by patient.

Implants were stratified by peri‐implantitis status and analyzed using two‐sample *t*‐tests for group comparisons and linear regression with an interaction term (Final mean PPD with Peri‐implantitis) to test whether the pocket depth‐bone loss relationship differed between healthy implants and those with peri‐implantitis.

The relationship between emergence angle and initial pocket depth was examined separately for each implant site (mesial, distal, buccal, and palatal/lingual). Simple linear regression models were fitted for each site with emergence angle as the predictor and initial pocket depth as the outcome variable.

Biological complications (peri‐implantitis and implant failure) were analyzed using logistic regression models, with emergence angles categorized as < 40°, between 40° and 59°, or ≥ 60°. Models were adjusted for smoking status and follow‐up duration. Statistical significance was set at *p* < 0.05.

For descriptive statistics, continuous variables are presented as means ± standard deviations with ranges, and categorical variables as frequencies and percentages.

## 3 Results

### 3.1 Sample Description

A total of 77 subjects where retrospectively screened. Out of these, 52 patients, with a total of 112 implants reached the 5‐year follow‐up, fulfilling the inclusion criteria. Five out of 52 included patients presented history of periodontitis (9.6%). Out of the 122 included implants, 8 where lost due to periimplantitis. Further details on the demographic of the included patients are reported in Table 1.

**TABLE 1:** Demographics of the included patients.

*N* Subjects (implants) screened	77 (154)
*N* Drop out subjects (implants)	25 (42)
*N* Subjects (implants) included	52 (112)
Age (mean; SD)	62.37 (11.5)
Gender (F/M)	36 (69.2%)/16 (30.8%)
Smoking (Y/N)	8 (15.4%)/44 (84.6%)
History of periodontitis (Y/N; %)	47 (90.4%)/5 (9.6%)
Follow Up in years (mean SD range)	6.6 (1.6) 5–11
*N* implant periimplantitis treatment	6 (5.4%)
Implant failure	8 (7.1%)

*Note:* 25 patients were excluded because they did not reach the 5‐year follow‐up.

### 3.2 Implant Distribution and Emergence Angles

**FIGURE 3:**
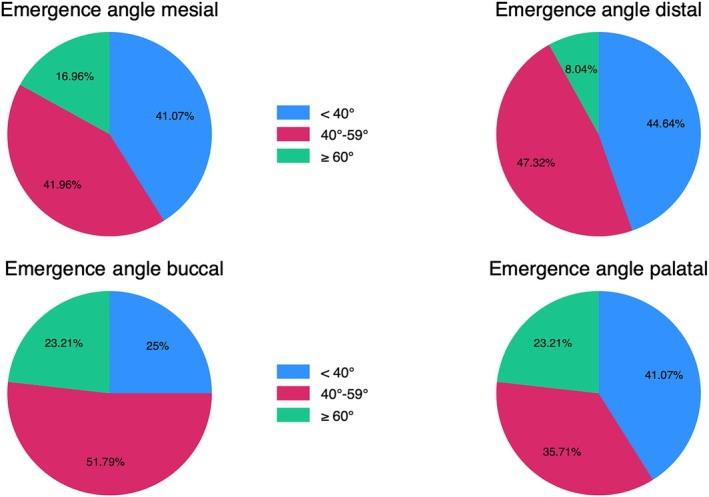
Distribution of prosthetic emergence angles divided into three categories: < 40°, between 40° and 59°, ≥ 60°.

**FIGURE A1:**
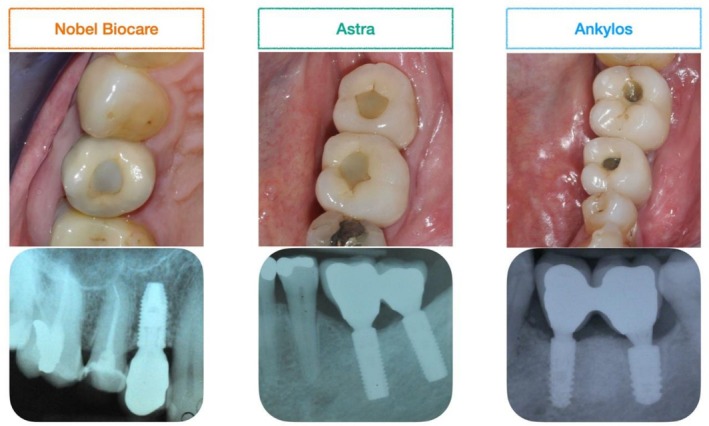
Clinical and radiographic representation of the three implant types included in this study.

All the patients were rehabilitated with bone level implants, and in detail 54.5% were Astra implants (Astra Tech, Dentsply, York, PA, USA); 41.0% were Nobel implants (Nobel Biocare, Switzerland) and 4.5% were Ankylos implants (Dentsply, York, PA, USA) (Figure A1). Further details on implant distribution are presented in Table 2. Looking at implant distribution, the implants were most frequently positioned in the lower molars (34.8%) and upper premolars (24.1%) as shown in Table 3. Mean emergence angles were: mesial (43.9° ± 14.7°), distal (40.6° ± 13.4°), buccal (49.5° ± 16.7°), and palatal (45.8° ± 18.9°) (Table 4). The buccal and palatal angles tended to be steeper (averaging around 46°–49°) compared to the mesial and distal angles (around 41°–44°). The distribution of abutment emergence angles after dividing them by three categories: < 40°, between 40° and 59°, ≥ 60° is depicted in Figure 3.

**TABLE 2:** Type of implants used.

	Number	%
Astra	61	54.5%
Nobel	46	41.0%
Ankylos	5	4.5%
Total	112	100%

**TABLE 3:** Implant position distribution.

Implant position		Frequency	Percent (%)
	11	2	1.8
	12	1	.9
	13	2	1.8
	14	8	7.1
	15	5	4.5
	16	6	5.4
	17	1	.9
	21	2	1.8
	22	2	1.8
	23	3	2.7
	24	5	4.5
	25	8	7.1
	26	8	7.1
	27	4	3.6
	34	4	3.6
	35	6	5.4
	36	17	15.2
	37	4	3.6
	44	2	1.8
	45	4	3.6
	46	15	13.4
	47	3	2.7
	Total	112	100.0

**TABLE 4:** Prosthetic emergence angles as measured on x‐rays (mesial and distal) or casts (buccal and lingual).

Angle (°)	Mean	SD	Min	Max
Mesial	43.9	(14.7)	15.0	86.0
Distal	40.6	(13.4)	14.0	71.0
Buccal	49.5	(16.7)	5.0	90.0
Palatal/Lingual	45.8	(18.9)	14.0	90.0

### 3.3 Overall Bone Loss and Pocket Depth

In general, the mean MBL and PPD values at different time points were quite small, mostly in the 0.1–0.8 mm range for bone loss and 1.8–3.9 mm range for pocket depths. This suggests that peri‐implant soft and hard tissues were generally stable and healthy in this sample (Tables 5, 6).

**TABLE 5:** Probing pocket depth as measured at different time points.

PPD [7]	Mean	SD	Min	Max
Loading				
Mesial	1.8	(1.5)	0	6
Distal	1.8	(1.5)	0	5
Buccal	1.5	(1.3)	0	5
Palatal/Lingual	1.4	(1.3)	0	6
1‐year				
Mesial	3.3	(1.4)	0	7
Distal	3.3	(1.4)	0	8
Buccal	2.8	(1.3)	0	6
Palatal/Lingual	2.4	(1.0)	0	5
Final examination				
Mesial	3.9	(1.9)	1	12
Distal	3.7	(2.1)	1	12
Buccal	3.2	(2.2)	1	15
Palatal/Lingual	3.0	(1.9)	1	10

**TABLE 6:** Marginal bone loss as measured at different time intervals.

MBL [7]	Mean	SD	Min	Max
Loading–1‐year				
Mesial	0.1	(0.5)	−2	2
Distal	0.1	(0.6)	−2	2
1 year–Final examination				
Mesial	0.7	(1.9)	−4	9.5
Distal	0.7	(1.8)	0	9
Loading–Final examination				
Mesial	0.7	(1.9)	−4	9
Distal	0.8	(2.0)	−2	9

There were no statistically significant differences between the angle groups for any variable or time point (*p*‐values > 0.05) (Table 7).

**TABLE 7:** Mean and standard deviation for marginal bone loss between different periods and for pocket depth at 1 year and at latest examination according to the corresponding abutment emergence angle.

	Corresponding emergence angle									
	< 40°			40°–59°			≥ 60°			*p*‐value
	Count	Mean	SD	Count	Mean	SD	Count	Mean	SD	
MBL mesial (loading‐1 year)	39	0.1	(0.5)	32	0.2	(0.5)	13	0.1	(0.8)	0.651
MBL mesial (loading‐latest)	45	0.8	(2.3)	46	0.4	(1.3)	19	1.2	(2.4)	0.322
MBL mesial (1 year‐latest)	38	0.9	(2.5)	32	0.4	(1.2)	13	0.7	(1.5)	0.465
MBL distal (loading‐1 year)	39	0.2	(0.5)	40	0.1	(0.7)	5	0.2	(0.8)	0.773
MBL distal (loading‐latest)	49	0.6	(2.0)	52	0.8	(1.8)	9	1.4	(2.8)	0.573
MBL distal (1 year‐latest)	38	0.7	(1.9)	40	0.8	(1.8)	5	0.9	(1.3)	0.920
PPD mesial loading	46	2.0	(1.8)	47	1.8	(1.4)	19	1.3	(0.9)	0.206
PPD mesial 1 year	39	3.3	(1.2)	32	3.2	(1.6)	13	3.2	(1.4)	0.934
PPD mesial latest	46	4.0	(2.4)	46	3.8	(1.3)	19	4.1	(1.9)	0.782
PPD distal loading	50	2.0	(1.6)	53	1.6	(1.3)	9	1.7	(1.7)	0.296
PPD distal 1 year	39	3.2	(1.2)	40	3.1	(1.3)	5	4.6	(2.6)	0.079
PPD distal latest	50	3.7	(2.3)	52	3.7	(1.7)	9	3.6	(3.3)	0.976
PPD buccal loading	28	1.4	(1.5)	58	1.8	(1.3)	26	1.0	(0.9)	0.038
PPD buccal 1 year	24	2.6	(1.2)	40	2.9	(1.4)	20	2.7	(1.4)	0.800
PPD buccal latest	28	3.0	(1.3)	58	3.5	(2.6)	25	2.6	(1.6)	0.165
PPD palatal/lingual loading	46	1.8	(1.3)	40	1.3	(1.3)	26	0.8	(0.9)	0.003
PPD palatal/lingual 1 year	36	2.6	(0.9)	28	2.5	(0.9)	20	2.1	(1.0)	0.130
PPD palatal/lingual latest	46	3.1	(2.0)	39	3.3	(2.1)	26	2.6	(1.7)	0.366

*Note:* ANOVA for differences between angle categories (Bonferroni).

Abbreviations: MBL, marginal bone loss; PPD, probing pocket depth; SD, Standard deviation.

The ≥ 60° group tended to have slightly higher mean MBL and PPD values compared to the < 40° group, but the small subgroup sizes and high variability (large SD) likely limited the power to detect significant differences.

### 3.4 Bone Loss, Emergence Angles and Pocket Depth

**FIGURE 4:**
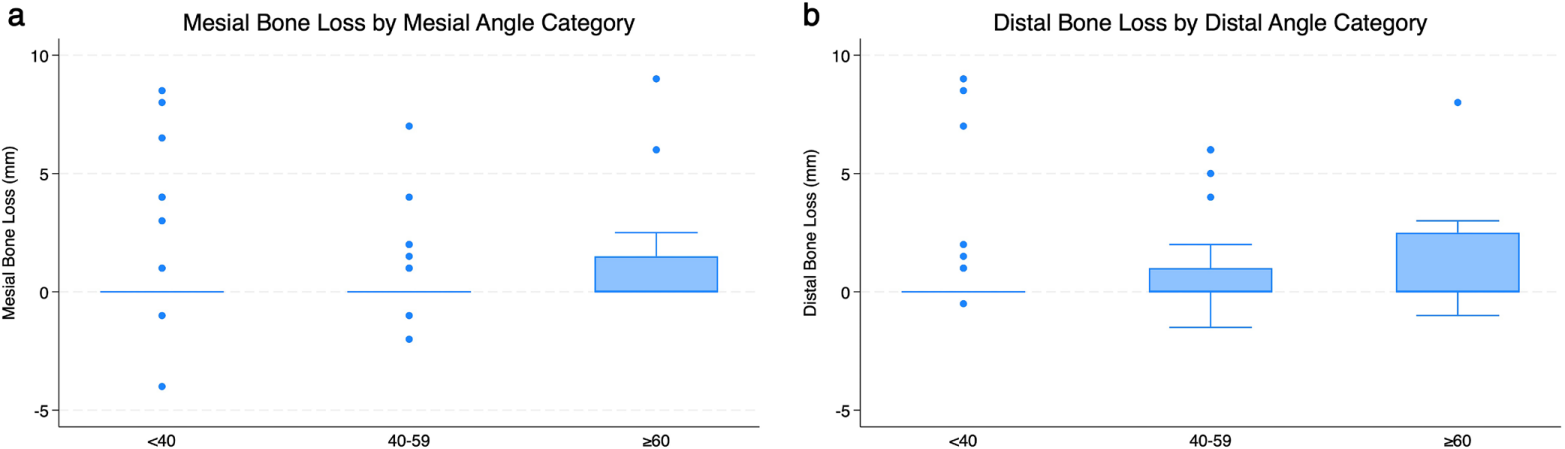
Box‐plot graphs reporting bone loss at final examination for each emergence angle category at mesial (a) and distal (b) site. No statistically significant difference was detected between the 3 groups (ANOVA test with Bonferroni compensation).

The distribution of marginal bone loss (MBL) at final examination according to emergence angle category is shown in Figure 4. No statistically significant difference was detected between the three groups, and also in the regression model controlling for age, gender, PPD, and smoking, with robust standard error estimation by accounting for patient clustering, no significant effect from angle category or continuous angle was detected (Tables A1 and A2).

**FIGURE 5:**
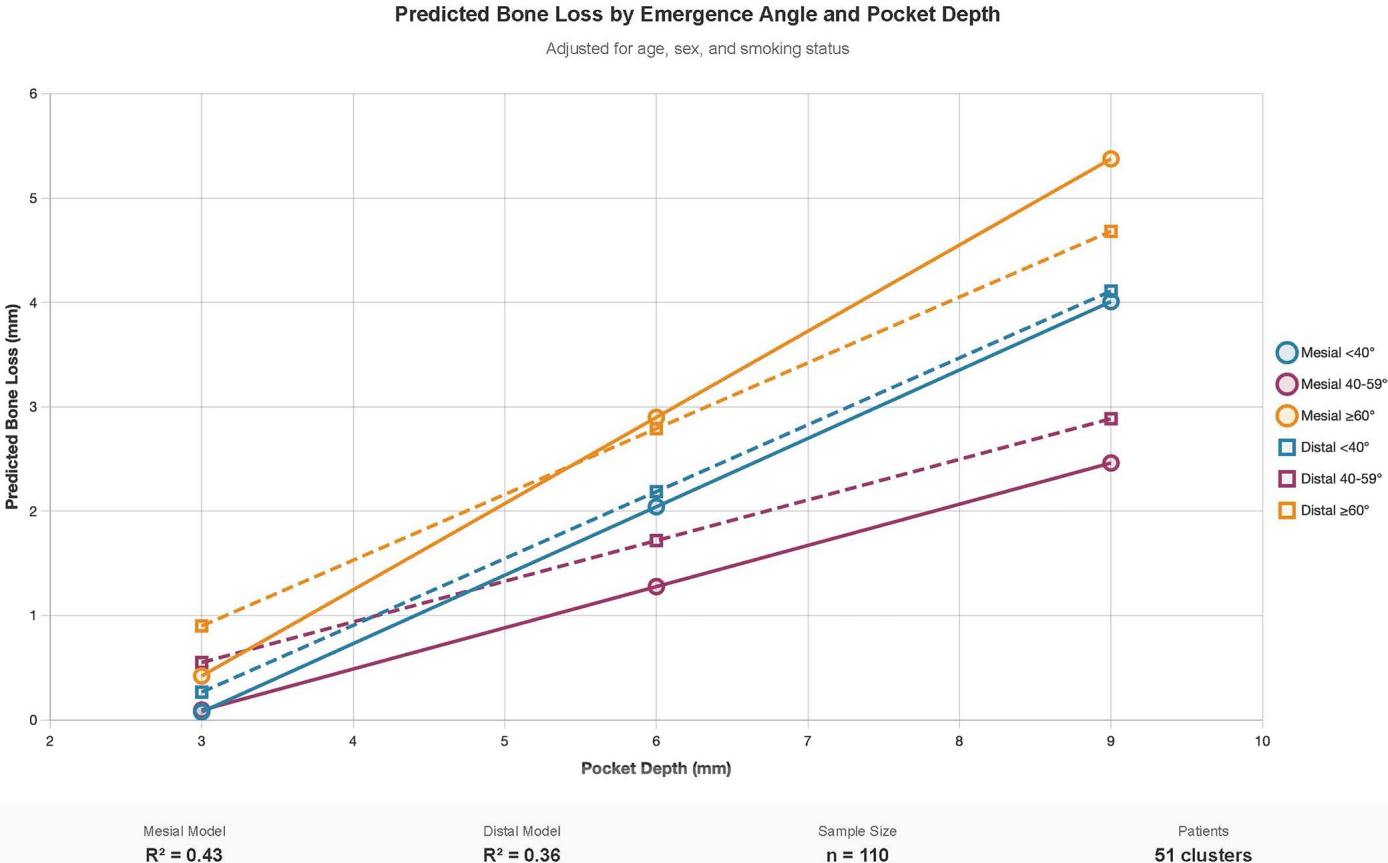
Predicted marginal effects of pocket depth on bone loss by emergence angle category. Solid lines represent mesial predictions; dashed lines represent distal predictions. Models adjusted for age, sex, and smoking status.

Pocket depth was the primary predictor of bone loss at both sites (mesial: *β* = 0.66, *p* < 0.001; distal: *β* = 0.64, *p* < 0.001), with no significant effects of emergence angle categories or patient covariates (mesial *R* ^2^ = 0.43, distal *R* ^2^ = 0.36) (Figure 5). All predictions showed increasing bone loss with greater pocket depth across emergence angle categories.

Years of follow‐up consistently showed a positive relationship with bone loss, suggesting a trend toward increasing bone loss over time, though again not reaching statistical significance.

**FIGURE 6:**
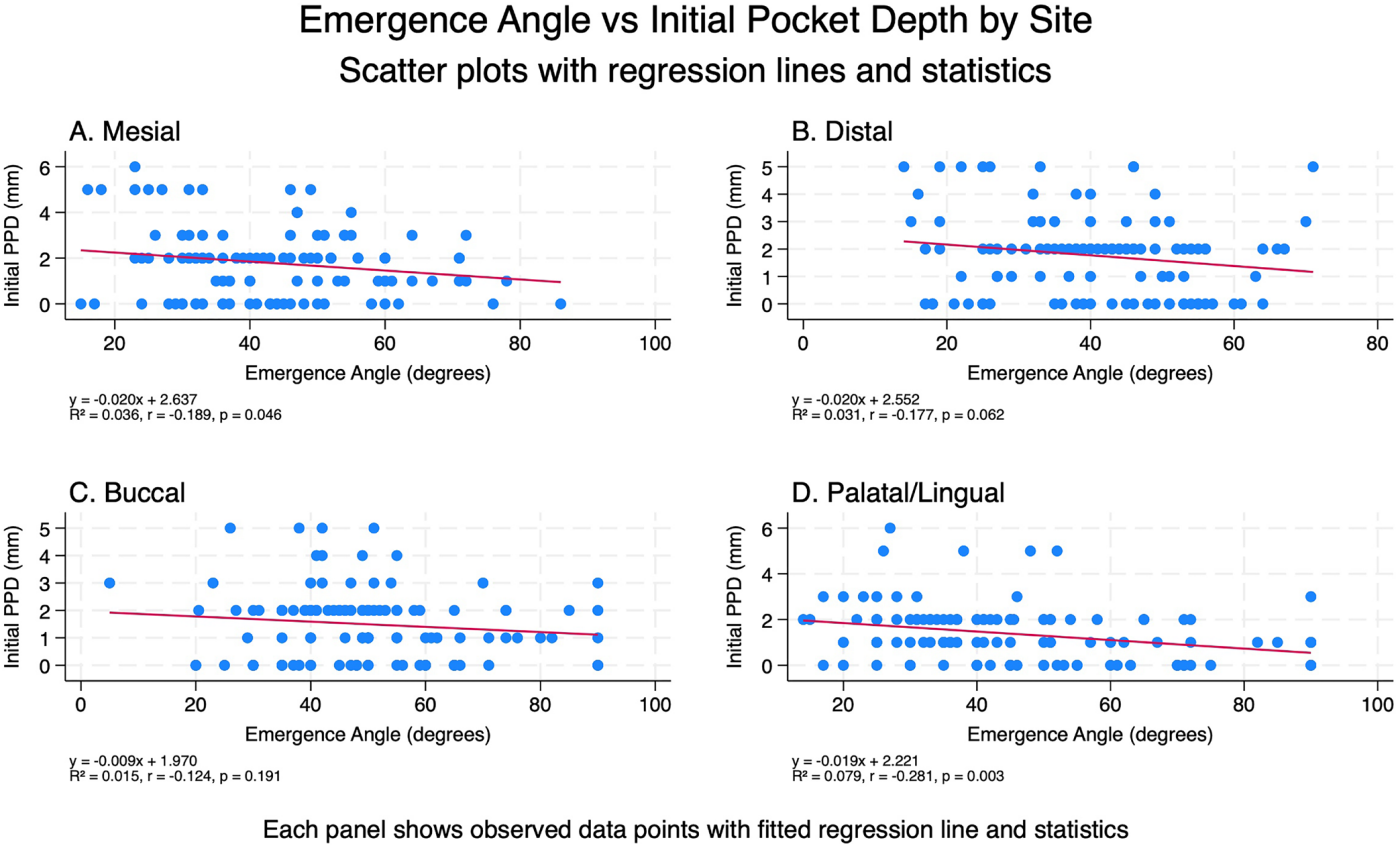
Simple linear regression models to examine the relationship between emergence angle and initial pocket depth at each implant site (mesial, distal, buccal, palatal/lingual), with results displayed as scatter plots with fitted regression lines, regression statistics (*R* ^2^, *r*, *p*‐value), and combined into a four‐panel comparative figure.

Emergence angle (Figure 6) showed weak negative correlations with initial pocket depth, reaching significance only at mesial (*p* = 0.046) and palatal/lingual sites (*p* = 0.003), with minimal explained variance across all sites (*R* ^2^ ≤ 0.079).

Six implants (5.5%) were diagnosed with peri‐implantitis during the follow‐up period. Implants with peri‐implantitis presented with significantly higher mean bone loss (2.25 ± 2.40 mm vs. 0.65 ± 1.86 mm, *p* = 0.047) and deeper mean pocket depths (5.0 ± 1.74 mm vs. 3.36 ± 1.77 mm, *p* = 0.019) compared to healthy implants. The relationship between pocket depth and bone loss differed significantly by peri‐implantitis status (interaction *p* = 0.001). In healthy implants, pocket depth measurements showed strong predictive value for bone loss, with each 1 mm increase in pocket depth corresponding to 0.79 mm of bone loss (*p* < 0.001, *R* ^2^ = 0.57). However, in implants with established peri‐implantitis, pocket depth measurements were not predictive of bone loss extent (*β* = −0.36, *p* = 0.622, *R* ^2^ = 0.07).

## 4 Discussion

This retrospective study aimed to evaluate the influence of iFDPs emergence angles on peri‐implant MBL and peri‐implantitis. Although trends were observed toward increased biological complications with wider prosthetic restorative angles, these associations did not reach statistical significance in the present sample.

The potential impact of prosthetic emergence angle on peri‐implant tissue stability has gained increasing attention in recent years, yet high‐quality clinical data remain scarce [19, 20]. Some clinical studies have suggested that restoration angles > 30° are associated with greater MBL [15, 16, 30]. However, this threshold was originally derived from a preclinical dog study on tooth‐supported crowns [31] raising questions about its validity in implant dentistry. More recently, clinical investigations have used higher thresholds, such as 40° [29] or 45° [32], based on the mean and median values of the included emergence angles, and therefore, allowing for a more meaningful comparison. Both these studies identified an increased risk of peri‐implantitis when these threshold values were exceeded [29, 32]. Supporting this threshold, a recent preclinical animal study [11] demonstrated radiographically and histologically that wide emergence angles (60°–80°) result in greater bone loss and disruption of the junctional epithelium compared to narrower angles (< 40°). On this basis, we applied 40° and 60° as reference thresholds in the present study.

Despite this background, we did not identify a significant correlation between wider emergence angles and MBL. One explanation may be the exclusive use of customized abutments. Unlike prefabricated Ti‐bases—commonly employed in previously published studies [15, 16, 17]—custom abutments enable individualized emergence profiles adapted to patient‐specific anatomical characteristics. Prefabricated Ti‐bases, by contrast, present the same emergence angle along the entire circumference, which may lead to lower anatomical adaptation and therefore less favorable peri‐implant tissue response. This interpretation aligns with another investigation reporting no association between MBL and emergence angle width when customized abutments with platform switching were used. Beyond emergence angle, customized abutments provide clinicians greater flexibility in emergence profile design, which has also been shown to enhance buccal hard‐ and soft‐tissue stability [33]. This concept has been reinforced by histologic evidence demonstrating that convergent abutment profiles promote connective tissue development in early healing phases compared to divergent designs [34].

Another novelty of this study is the assessment of emergence angles not only on the mesial and distal aspects, as typically reported, but also on the buccal and palatal/lingual sides. Emergence angles were slightly wider on the buccal aspect (49.9°) compared with mesial (43.9°) and distal (40.6°) aspects. This is important, since the buccal side is particularly relevant both aesthetically and biologically, as it is considered more prone to MBL in cases of wide angles [11]. To examine the buccal aspect, the role of PD as a predictor factor of MBL was investigated.

Interestingly, the marginal effects analysis demonstrated that pocket depth was the primary predictor of bone loss at both mesial and distal sites, with a consistent relationship across emergence angle categories (0.64–0.66 mm bone loss per 1 mm pocket depth increase). While emergence angles showed no significant effects, the robust pocket depth‐bone loss relationship (mesial *R* ^2^ = 0.43, distal *R* ^2^ = 0.36) supports the clinical utility of systematic pocket probing for quantifying bone loss extent in implant monitoring protocols, independent of implant positioning characteristics [35].

Pocket depth was a predictor of the magnitude of bone loss for healthy implants, but not for implants with peri‐implantitis. These findings suggest that pocket depth measurements are most valuable as a diagnostic tool for detecting early bone loss in healthy implants, where deeper pockets reliably indicate greater bone loss. Once peri‐implantitis is established, the extent of bone loss becomes less predictable from pocket depth alone, likely reflecting the complex inflammatory process that characterizes this condition. This supports the clinical utility of pocket depth measurements for early detection and monitoring of peri‐implant health, rather than as predictors of bone loss severity in already diseased implants. In other words, in the case of peri‐implantitis, progressive radiographic MBL and clinical signs of inflammation play a major diagnostic role [35]. The weak correlations suggest that emergence angle has limited influence on initial pocket depth measurements, with site‐specific anatomical factors potentially playing a more important role than implant positioning characteristics in determining baseline probing depths.

This study has some limitations. Its retrospective design inherently limits causal inference, and residual confounding cannot be excluded. Although the sample size was adequate for preliminary exploration, it may have been underpowered to detect subtle but clinically relevant differences between groups. Another limitation of the present study is that buccal and palatal emergence angle measurements were based on standardized photographs of the implant restorations positioned on analogs, which may have limited the reproducibility of the exact measurements. It must be considered that at the time of the investigation, advanced techniques such as intraoral or extraoral digital scanning, which could improve measurement accuracy, were not yet widely implemented or validated.

Moreover, as all prostheses were supported by customized abutments, the findings may not be directly generalizable to cases rehabilitated with prefabricated Ti‐bases.

## 5 Conclusion

Within the limitations of this retrospective analysis, no significant association was observed between prosthetic emergence angle and peri‐implant marginal bone loss or peri‐implantitis when customized abutments were used. Pocket depth appeared as the most consistent predictor of bone loss in healthy implants. While these findings suggest that individualized abutment design may help mitigate some risks associated with wide emergence angles, the results should be interpreted with caution given the study's retrospective design, limited sample size, and potential measurement variability. Future prospective studies with larger cohorts and long‐term follow‐up are needed to confirm these observations and further clarify the relationship between prosthetic design, peri‐implant tissue health, and aesthetic outcomes.

## Funding

The authors have nothing to report.

## Conflicts of Interest

The authors declare no conflicts of interest.

## Data Availability

The data that support the findings of this study are available from the corresponding author upon reasonable request.

## Appendix A

### Regression Models

**TABLE A1:** Multivariable regression analysis with pocket depth × emergence angle interaction predicting mesial (a) and distal (b) bone loss. Coefficients show change in bone loss [7] per unit increase or vs. reference categories (< 40° angle, female, non‐smoker). Interaction terms indicate differential pocket depth effects across angle categories. Standard errors clustered by patient (*n* = 110, 51 clusters).

(a) Marginal bone loss mesial (Loading—Final ex.)	Coefficient	Robust std. err.	*p*‐value
PPD mesial at final examination	0.650	1.272	< 0.0001
Mesial angle (< 40° ref)			
40–59	0.992	0.709	0.168
≥ 60	−0.525	1.259	0.678
Mesial angle cat * PPD mesial			
40–59	−0.309	0.210	0.147
≥ 60	0.265	0.323	0.415
Age	0.019	0.013	0.171
Gender (ref. female)			
Male	0.106	0.343	0.759
Smoker (ref. no)			
Yes	−0.189	0.314	0.551
Follow‐up (years)	0.134	0.098	0.177
Cons	−3.954	1.159	0.001

(b) Marginal bone loss distal (loading–final ex.)	Coefficient	Robust std. err.	*p*‐value
PPD distal at final examination	0.628	1.684	< 0.0001
Distal angle (< 40° ref)			
40–59	0.946	0.830	0.260
≥ 60	0.403	1.206	0.740
Distal angle cat * PPD distal			
40–59	−0.234	0.233	0.322
≥ 60	0.054	0.237	0.822
Age	0.008	0.018	0.691
Gender (ref. female)			
Male	0.441	0.456	0.338
Smoker (ref. No)			
Yes	−0.409	0.430	0.346
Follow‐up (years)	0.134	0.124	0.283
Cons	−2.993	1.450	0.044

**TABLE A2:** Multivariable regression analysis of mesial (a) and distal (b) bone loss predictors. Coefficients show change in bone loss [7] per unit increase for continuous variables or vs. reference category (female, non‐smoker). Standard errors clustered by patient (*n* = 110, 51 clusters).

(a) Marginal bone loss mesial (loading‐final ex.)	Coefficient	Robust std. err.	*p*‐value
PPD mesial at final examination	0.641	0.122	< 0.0001
Mesial angle (°)	0.011	0.012	0.369
Age (years)	0.170	0.139	0.226
Gender (ref. female)			
Male	0.260	0.363	0.477
Smoker (Ref. No)			
Yes	−0.237	0.327	0.472
Follow‐up (Years)	0.071	0.110	0.525
Cons	−3.873	1.404	0.008

(b) Marginal bone loss distal (loading‐final ex.)	Coefficient	Robust std. err.	*p*‐value
PPD distal at final examination	0.562	0.115	< 0.0001
Distal angle (degrees)	0.010	0.013	0.445
Age (years)	0.009	0.017	0.604
Gender (ref. female)			
Male	0.570	0.450	0.211
Smoker (Ref. No)			
Yes	−0.329	0.368	0.375
Follow‐up (years)	0.132	0.116	0.261
Cons	−3.182	1.562	0.047
